# A novel *Bartonella*-like bacterium forms an interdependent mutualistic symbiosis with its host, the stored-product mite *Tyrophagus putrescentiae*

**DOI:** 10.1128/msystems.00829-23

**Published:** 2024-02-21

**Authors:** Qing Xiong, Bruno Sopko, Pavel B. Klimov, Jan Hubert

**Affiliations:** 1 School of Biomedical Sciences, The Chinese University of Hong Kong, Hong Kong, China; 2 Department of Health Technology and Informatics, The Hong Kong Polytechnic University, Hong Kong, China; 3 Crop Research Institute, Prague, Czechia; 4 Purdue University, Lilly Hall of Life Sciences, West Lafayette, Indiana, USA; 5 Department of Microbiology, Nutrition and Dietetics, Faculty of Agrobiology, Food and Natural Resources, Czech University of Life Sciences Prague, Prague, Czechia; Chinese Academy of Sciences, Beijing, China

**Keywords:** *Bartonella*, mite, ants, symbionts, vitamin, nutrition, house dust, stored-product

## Abstract

A novel *Bartonella*-like symbiont (BLS) of *Tyrophagus putrescentiae* was characterized. BLS formed a separate cluster from the *Bartonella* clade together with an ant symbiont. BLS was present in mite bodies (103 16S DNA copies/mite) and feces but was absent in eggs. This indicated the presence of the BLS in mite guts. The BLS showed a reduction in genome size (1.6 Mb) and indicates gene loss compared to *Bartonella apis*. The BLS can be interacted with its host by using host metabolic pathways (e.g., the histidine and arginine metabolic pathways) as well as by providing its own metabolic pathways (pantothenate and lipoic acid) to the host, suggesting the existence of a mutualistic association. Our experimental data further confirmed these potential mutualistic nutritional associations, as cultures of *T. putrescentiae* with low BLS abundance showed the strongest response after the addition of vitamins. Despite developing an arguably tight dependency on its host, the BLS has probably retained flagellar mobility, as evidenced by the 32 proteins enriched in KEGG pathways associated with flagellar assembly or chemotaxis (e.g., fliC, flgE, and flgK, as highly expressed genes). Some of these proteins probably also facilitate adhesion to host gut cells. The microcin C transporter was identified in the BLS, suggesting that microcin C may be used in competition with other gut bacteria. The 16S DNA sequence comparison indicated a mite clade of BLSs with a broad host range, including house dust and stored-product mites. Our phylogenomic analyses identified a unique lineage of arachnid specific BLSs in mites and scorpions.

**IMPORTANCE:**

A *Bartonella*-like symbiont was found in an astigmatid mite of allergenic importance. We assembled the genome of the bacterium from metagenomes of different stored-product mite (*T. putrescentiae*) cultures. The bacterium provides pantothenate and lipoic acid to the mite host. The vitamin supply explains the changes in the relative abundance of BLSs in *T. putrescentiae* as the microbiome response to nutritional or pesticide stress, as observed previously. The phylogenomic analyses of available 16S DNA sequences originating from mite, scorpion, and insect samples identified a unique lineage of arachnid specific forming large *Bartonella* clade. BLSs associated with mites and a scorpion. The *Bartonella* clade included the previously described *Ca*. Tokpelaia symbionts of ants.

## INTRODUCTION

*Bartonella* includes fastidious, gram-negative, facultative intracellular pathogens (1). Parasitic *Bartonella* species usually exist in two specific habitats: the gut of obligately bloodsucking arthropod vectors, where they are exposed to toxic concentrations of heme, and the bloodstream of mammalian hosts, where they are deprived of access to heme and iron (1). A recent hypothesis suggested that the mammalian pathogens of the genus *Bartonella* originated from an insect-associated gut symbiont in contrast with the evolution of other vector-borne pathogens (2). Insight into the genomes of insect symbionts is important for understanding their importance to the host and the evolution of this group.

The insect symbionts include bee and ants. *Bartonella apis* lives in the gut of *Apis mellifera* honeybees (3), probably as an extracellular symbiont (2). This and other species, *Bartonella choladocola* and *Bartonella apihabitans*, from the bee gut form a monophyletic clade together with parasitic *Bartonella tamiae* (4). This represents the ancestral condition for *Bartonella*, as inhabiting the gut of blood-feeding insects occurred prior to inhabiting the mammalian bloodstream (2). The other lineages include monophyletic lineage of ant symbionts identified in herbivorous ants belonged to *Bartonella* clade. The ants symbionts contribute to dietary switch from predatory to herbivory in ants is associated with these symbionts [e.g., *Procryptocerus* (5), *Cephalotes* (5), leaf-cutting ants *Acromyrmex* (6)]. However, the symbionts live inside the gut of the myrmecophyte ants of the genus *Tetraponera* (7), carnivorous ants (e.g., *Terataner*) (8–10), and scavenger *Dolichoderus* ants (11). The genomes of these ant symbionts cluster outside the *B. apis* group (2, 5). Although ant-related taxa show losses of many metabolic pathways (e.g., essential amino acid biosynthesis in *Candidatus* Tokpelaia from *Acromyrmex*), they are fully dependent on their hosts (6).

In addition to the symbionts in insects, there is evidence of the next possible lineage of *Bartonella*-like symbionts (BLSs) in mites. This evidence is based on 16S RNA cloning and sequencing of the clones of stored-product mites (*Acarus siro*, *Tyrophagus putrescentiae*, and *Carpoglyphus lactis*) (12–14), house dust mites (*Dermatophagoides farinae*) (15), and blood-feeding parasitic mites *Dermanyssus galinae* (16). Phylogenetic analyses indicated the existence of a symbiont clade outside of *B. apis* and an ant symbiont (12, 16). Although the BLS was reported in the honeybee parasite *Varroa destructor* (17), the 16S RNA sequences revealed that it was *B. apis*. This parasite could have originated from the mite diet, i.e., host hemolymph (17). While a few 16S RNA analyses have been conducted, a comparison of mite-related clades is lacking, and no genome from this symbiont clade has been sequenced yet.

Stored-product and house dust mites can survive in house dust, including human dander and nails and the microorganisms growing on them, as well as in various stored foods, such as wheat, cheeses, dried ham, and animal feed (18, 19). Such habitats and food sources lead to dietary vitamin and nitrogen imbalance, and interactions with gut symbiotic microorganisms were expected. Indeed, BLSs had from 20% to 30% bacterial 16S DNA reads in the microbiome of the stored-product mite *T. putrescentiae* body and fecal fractions (SPGM) (20). Stored-product and house dust mites are allergen producers (21), and the allergens they produce include compounds biochemically known to be produced in response to bacteria (22–24). In this manner, mite symbiotic bacteria indirectly influence allergen production (22) and contamination of the human environment. However, the underlying mechanisms are still poorly understood.

The profiles of 16S RNA sequences from BLS of *T. putrescentiae* samples showed changes correlated with mite diet perturbations or the response to pesticide treatment, resulting in an increase in its relative abundance in the microbiome (25–28). Similarly, the profiles of the *B. apis* group responded immediately to pesticide treatment (29) or parasite stress (30) in the honeybee microbiome. This raises the question of whether BLSs are biologically important to their mite hosts.

In this study, we used Illumina and PacBio sequences of *T. putrescentiae* metagenome samples to assemble the genome of BLS. The genome of BLS was used to establish the BLS phylogenetic position using the comparison of the whole genomes and/or 16S RNA. We employed BLS-16S DNA specific primers to quantify the proportion of symbiont infestation in the mites and the numbers of symbionts in the mite body, fecal fraction (spent growth medium: SPGM), and mite eggs. We establish numbers of BLS reads from meta-transcriptome samples of five *T. putrescentiae* cultures. Based on correlation analyses, we identify symbiont and host metabolic pathways and identified symbiont metabolites that could be beneficial to the mite host and vice versa.

## RESULTS

**Fig 1:**
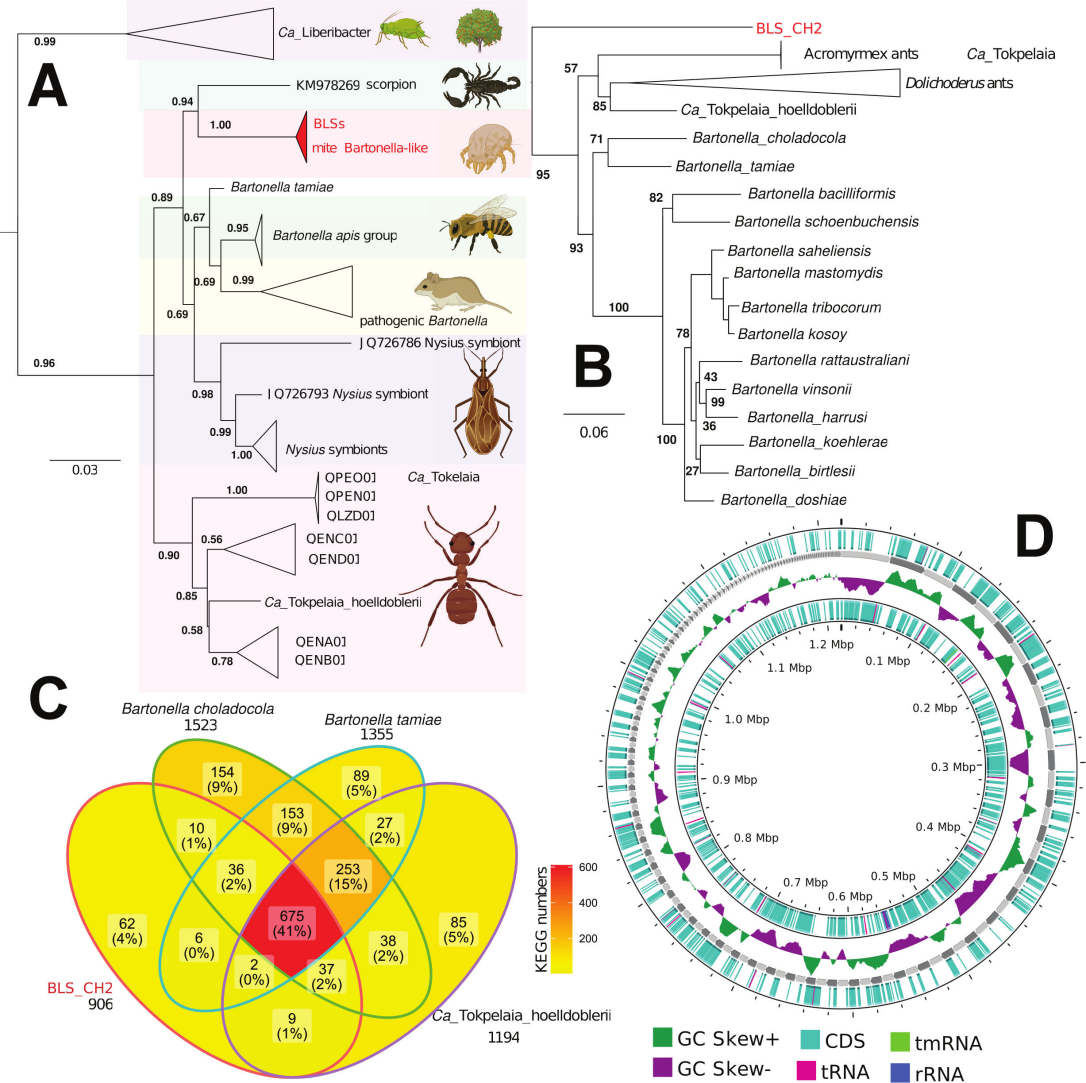
Phylogenomic affinities and genomic features of the *Bartonella*-like symbiont associated with the stored-product mite *Tyrophagus putrescentiae*. (**A**) Phylogenetic tree; the GTR + G + I nucleotide substitution model based on 16S RNA. (**B**) Maximum likelihood phylogeny based on orthologous protein groups, setting the M1CR0B1AL1Z3R data set outgroup to *Brevundimonas halotolerans*. (**C**) The overlapping of predicted KEGG genes in select bacterial species. (**D**) Genomic characteristics of the novel BLS (BLS_CH2). See Table S8 for list of sequences or genomes used for analyses.

The assembly of different genome sequences of BLSs obtained from *T. putrescentiae* cultures was performed as follows (Table 1): (i) **BLS_CH1** single-contig annotation of Chinese mites; (ii) **BLS_CH2** multiple-contig annotation of Chinese mites; (ii) **BLS_5** multiple-contig annotation of a mix of *T. putrescentiae* cultures; and (iv) **BLS_5S** multiple-contig annotation of a 5 S *T*. *putrescentiae* culture (Table S1). The genome size ranged from 1.15 to 1.37 Mb, and the GC content ranged from 39.8% to 40.73%. The completeness of both genomes ranged from 79% to 91%, as estimated by BUSCO using the Alphaproteobacteria database (31) (Table S2). The MASH average nucleotide identity of the assembled genomes was almost 99% (Fig. S1). The number of predicted proteins ranged from 1084 (BLS_5) to 1269 (BLSCH_2). The PHMMER pairwise comparison of the proteins showed that all genome assemblies shared between 907 and 929 proteins, while the number of unique proteins was up to 94 (7%) in BLS_CH2. The genomes shared 678 proteins annotated to KEGG pathways, and 23 KEGG-assigned proteins were unique to BLS_CH2 (Fig. S1). In comparison to *Bartonella* clade genomes (Fig. 1B), the number of open reading frames (ORFs) of BLSs was below the median, while that in the gut contents reached median values (Fig. S2). The MASH clustering showed that BLS symbionts formed separate taxa outside the *Bartonella* genus and *Ca*. Tokpelaia (Fig. S3). The assembled BLSs genomes covered 17 to 27 KEGG modules (Table S2).

**TABLE 1:** Variability in the *Bartonella*-mite expression in six *Tyrophagus putrescentiae* cultures^*a*^

Distance		Robust Aitchison		Jaccard	
	Expression	KEGG	genes	KEGG	genes
	R^2^	0.507	0.487	0.725	0.682
	F	7.219	7.292	6.646	9.178
	P	<0.001	<0.001	<0.001	<0.001

Culture	Df	F	P	F	P
5N	1	20.845	<0.001	27.521	<0.001
5P	1	16.095	<0.001	31.6812	<0.001
5K	1	23.965	<0.001	38.2513	<0.001
5S	1	5.981	0.009	5.812	0.003
5Tk	1	2.748	0.204	4.059	0.012
5L	1	3.92	0.065	3.532	0.014
Residual	42				

^*a*^ We used a distance-based redundancy analysis (dbRDA) to build several models: KEGG genes, all genes with two types of distances: Robust Aitchison and Jaccard (presence/absence based). The importance of the 'mite culture' variable was tested using forward selection; *P*-values are based on a permutational test.

The BLS was inferred as sister to an undescribed *Bartonella* species from a scorpion midgut (Fig. 1A). This group (mite and scorpion associated) was sister to the group comprising blood-feeding insect symbionts, honeybee gut symbionts (*B. apis* group), the human blood pathogen *B. tamiae*, and mammal blood pathogens of the genus *Bartonella* (Fig. 1A). This entire lineage (the BLS from mites plus the others mentioned above) was sister to the ant symbionts of *Ca*. Tokpelaia (Fig. 1A). Our alternative topology, based on the complete genome and fewer taxa, inferred BLS as sister to *Ca*. Tokpelaia and all other *Bartonella*, i.e., in the basal position of the tree (Fig. 1B).

In the comparison of selected genomes of *Bartonella* clade (listed in Fig. 1B), the BLS_CH2 had a GC content of approximately the median level (median = 40.5%, BLS_CH2 = 40.73), while the ORF counts were below the median (median = 1693; BLS = 1235) (Fig. S2), indicating substantial gene loss. The protein comparison (BLS_CH2) of related taxa from *Bartonella* clade (Fig. S4) showed the following features: BLS_CH2 has the highest numbers of similar proteins (59%) to *B. tamiae*, *B. choladocola*, *Ca*. *T. hoelldoblerii*. The BLS shared the most genes (69%) with *B. tamiae*, followed by *B. choladocola,* with 67% shared genes (Fig. S5A). The next comparison indicated that BLS shared 57% to *Acromyrmex* leaf-cutting ant hosts (6). The lowest number of shared protein (45%) among BLS and symbionts of *Dolichoderus* ants (11). According to this analysis, BLS_CH2 had 31%–42% unique proteins. The pool of shared proteins was almost identical for all compared taxa (Fig. S5B). Based on the genes predicted by KEGG analysis, the BLS shared 47% of its genes with *B. choladocola*, 43% with *B. tamiae,* and 44% with *Ca*. *T. hoelldoblerii* (Fig. 1).

The abundance of the BLS was analyzed in seven host cultures (5K, 5N, 5P, 5L, 5Pi, 5S, and 5Tk) based on the 16S RNA sequence and numbers of reads in transcriptome samples (Table 2). PCRs with specific primers for *Bartonella* 16S RNA gene fragment revealed that the proportion of *T. putrescentiae* mites inhabited by the BLS ranged from 56% (culture 5Pi) to 97% (5P) (Table S5). Using qPCR with specific *Bartonella* primers, we did not identify the BLS in the 5L and 5S cultures (Fig. S5). There were more than 16S copies per 10^3^ mites, except for the 5K culture, which had a 10-fold lower abundance of this symbiont (Fig. S5). qPCR confirmed the presence of BLSs in the mite body homogenates and fecal fraction (SPGM) but not in the eggs (Table S9). In the SPGM, the number of copies was approximately 10^6^ per g, except for cultures 5Pi and 5Tk, where this value was up to tenfold lower (Fig. S5). BLS abundance varied among the cultures (Table S10), and 5P, 5N, and 5K had higher abundance of BLSs than the remaining cultures based on expression analysis (Fig. S2). The source of the mite culture (i.e., the variable *mite culture*) was a significant factor influencing gene expression in the BLS (dbRA: df = 6.42 *F* = 6.37, *P* = 0.001; *R*^2^ = 0.48).

**TABLE 2:** The list of *Tyrophagus putrescentiae* cultures and their origin, the table shows the samples for genome and transcriptome and PCR analyses^*a*^

Mite culture	Name	Origin	Collector	Date	Diet	Samples genome				Sample transcriptome	Sample	
						BLS_5	BLS_5S	BLS_CH1	BLS_CH2		PCR	qPCR
5K	Koppert	Laboratory, the Netherlands	E. Baal	2012	Grain-derived diet	X				X	X	X
5L	Laboratory	Grain stoore, Bustehrad, Czechia	E. Zdarkova	1996	Grain-derived diet	X				X	X	X
5N	Nestle	food store, USA	J. Hubert	2007	Dog kernel diet	X				X	X	X
5P	Phillips	Laboratory, USA	T. W. Phillips	2014	Dog kernel diet	X				X	X	X
5Pi	Biscuit	Biscuit contamination, Prague, Czechia	M. Nesvorna	2015	Grain-derived diet	X				X	X	X
5S	Dried ham	Food factory, Cesena, Italy	A. Sala	2013	Grain-derived diet	X	X			X	X	X
5Tk	Teplice	Horse feed contamiantion, feed store, Teplice, Czechia	M. Nesvorna	2015	Grain-derived diet	X						
CH	Chinese	Laboratory, China	Zhi-Gang Liu	2017	Grain-derived diet			X	X			

^*a*^ For genome analyses, the genome assemblage BLS_5, BLS_5S, BLS_CH1, BLS_CH2 are showed and X means that the culture metagenome was utilized in the assembalge, for the rest of samples X means that samples were tested indepdendantly in 6 or 7 replicates. The details sample characteristic are provided in Supplementary dataset Table S1.

Species of *Bartonella* have type IV and V secretion systems that are used for the infection of endothelial cells and erythrocytes in mammalian hosts (11, 32, 33). These systems were not present in the BLSs (our data). Although BLS protein sequences were assigned to type IV secretion system protein *VirD4* (K03205) (Table S12) with a low score (Tables S4 to S6), the HMMER search did not confirm these results. The *Sec* translocase and the signal recognition particle pathway (SecSRP) were complete except for secretion monitor *secM* (K13301) in the BLS (Table S12).

**Fig 2:**
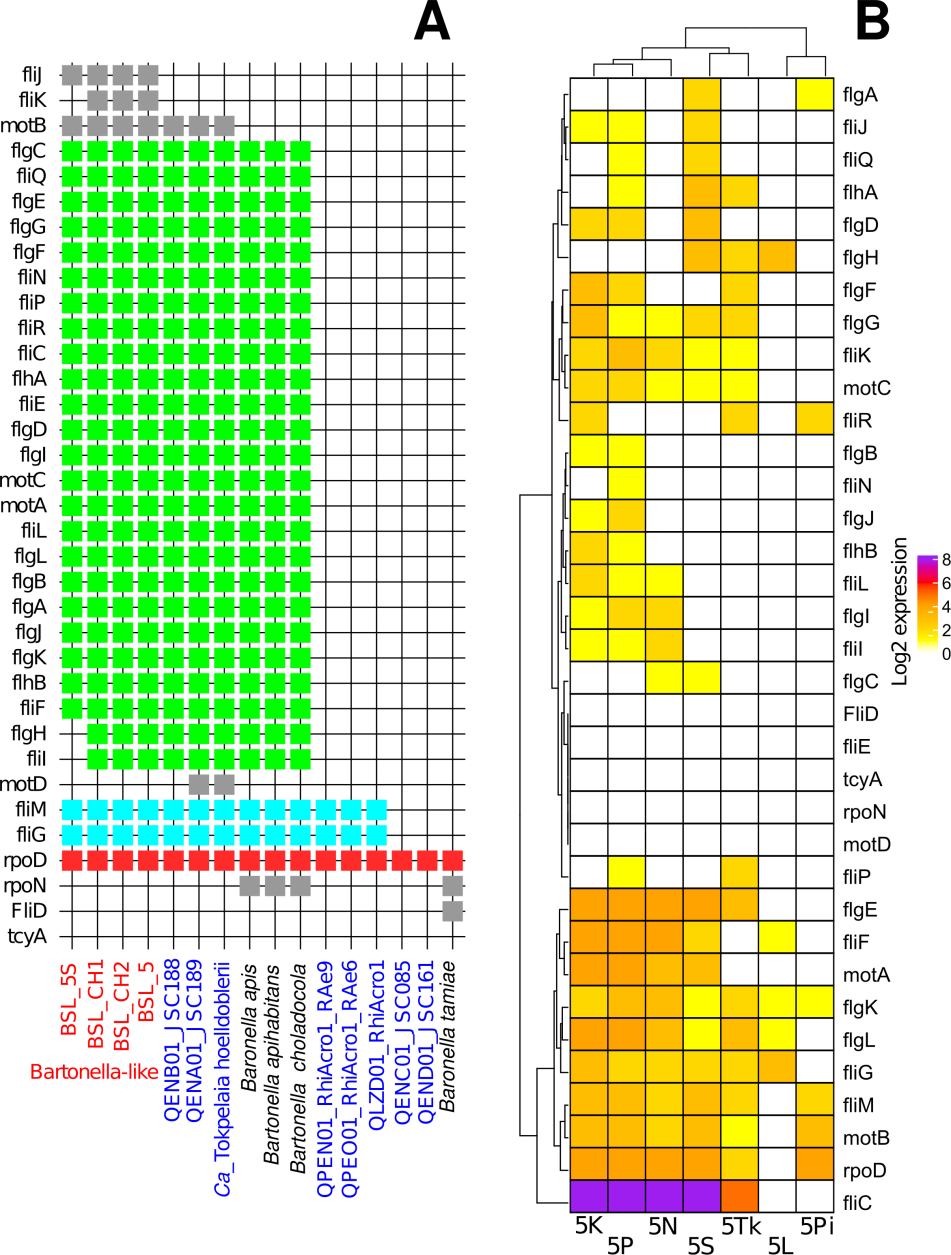
KEGG genes associated with flagellar assembly and chemotaxis (**A**) and their expression in different mite cultures (**B**). (**A**) Gene presence/absence in the *Bartonella*-like symbiont and related taxa based on seriation; different gene groups are color-coded; different isolation sources of *Ca*. Tokpelaia are indicated by blue colors in the labels. (**B**) Gene expression in the *Bartonella*-like symbiont from seven mite cultures.

A flagellar assembly is present in free-living Rhizobiales (ancestors of Bartonellaceae), which have a set of genes encoding functional flagella. Pathogenic *Bartonella* species use their flagellum to invade erythrocytes although flagellum loss has occurred quite frequently in this group (6). The unknown is situation in the symbiotic bacteria from *Bartonella* clade. In the BLS, by KEGG analysis, we identified 32 genes associated with flagellar assembly or chemotaxis, indicating the flagellar mobility of the symbiont and possible adhesion to host gut cells (Fig. 2A). Of these genes, *fliC*, *flgE*, and *flgK* had elevated expressions (Fig. 2B). Drastic loss of flagellar genes occurred in some *Ca*. Tokpelaia strains (6), such as RhiAcro1, RhiAcro1-RAEe6, RhiAcro1_Rae9, JSC161, and JSC085 (Fig. 2A), while *Ca*. *T. hoelldoblerii* (JSC188, JSC189) exhibited nearly complete gene sets (Fig. 2A), analogous to the BLS. Massive gene losses were observed in the *B. tamiae* group compared to its sister group, the *B. apis* group (Fig. 2A).

The outer membrane channel *TolC* (K12340) is involved in the export of small molecules and toxins across the outer membrane of gram-negative bacteria, including *Ca*. Tokpelaia and *Bartonella* (9). Here, we recovered three clusters of *TolC* proteins, with several species having two copies (e.g., *B. tamiae*, *B. apis*), while the BLS and some *Ca*. Tokpelaia strains had only one copy (Fig. S6). Genes in different clusters differed by the lengths of signal peptides and positions of two outer membrane efflux protein domains.

**Fig 3:**
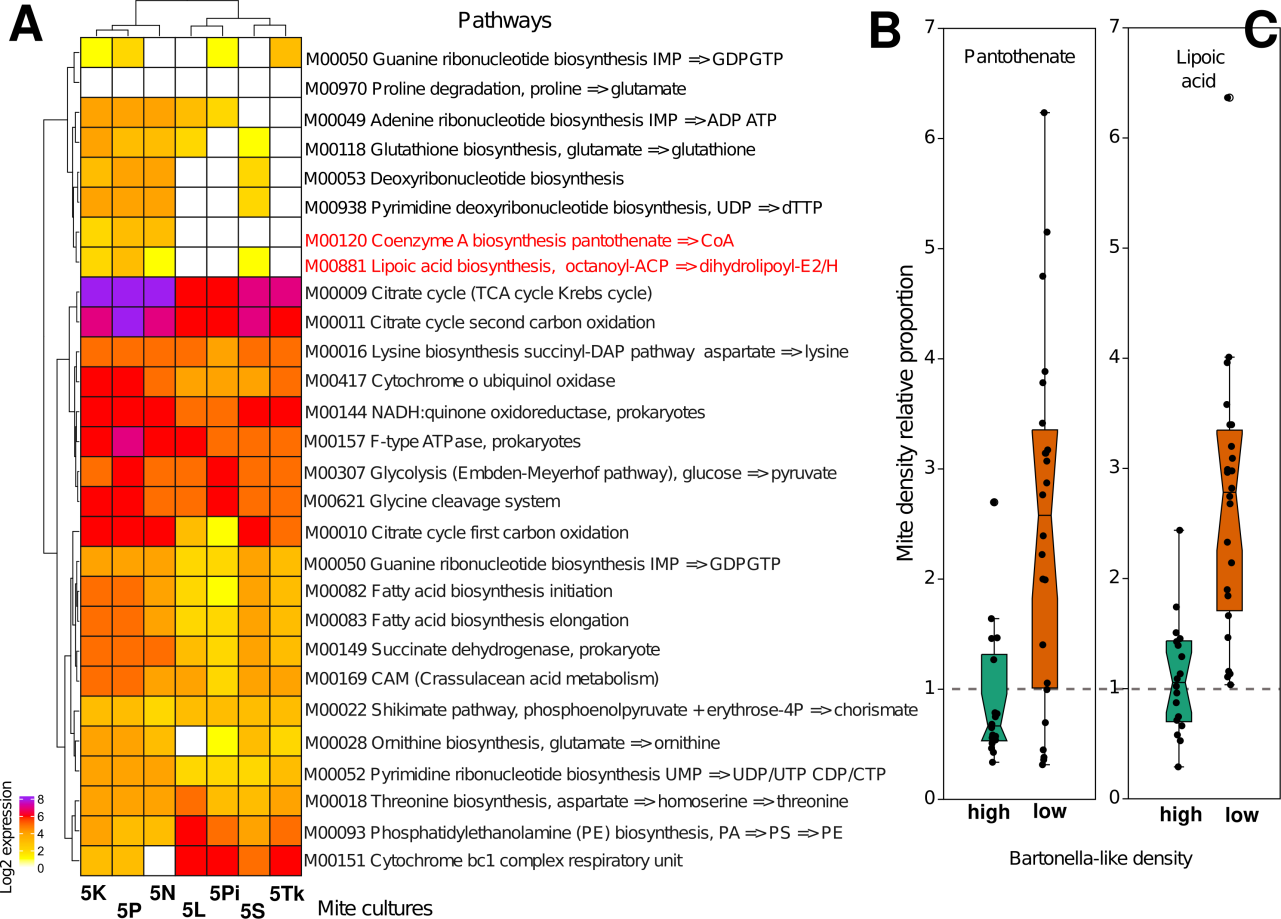
(**A**) Gene expression of 22 complete metabolic pathways of the *Bartonella*-like bacterial symbiont from seven different mite cultures. Values are shown as the log2 sum of reads per mite culture. (**B and C**) Growth of *T. putrescentiae* cultures with low and high densities of *Bartonella*-like symbionts on pantothenate (**B**) and lipoic acid (**C**). Mite population growth was calculated by dividing the value for the vitamin diet to that for control without vitamin for each mite culture. The mean population density for cultures with low (5S, 5Pi, 5Tk, and 5L) and high (5P, 5N, and 5K) density are shown (see Table 2 for mite culture description).

The pantothenate and lipoic acid synthesis pathways are vitamin synthesis pathways that were present in the BLS (Fig. 3A), while the riboflavin pathway was missing. The addition of lipoic acid and pantothenate to the diet showed a larger positive effect on the growth and reproduction of the mite host, *T. putrescentiae,* in cultures with a relatively low abundance of BLSs (Table S14): 2.6- and 1.6-fold increase, respectively (Mann–Whitney test; pantothenate: *U* = 94, *P* = 0.002; folic acid: *U* = 40.5, *P* = 0.001 (Fig. 3B).

The biotin synthesis pathway is missing in the BLS, while biotin transporters (*bioY*, *bioN,* and *bioM*) are present (Table S12). This indicates that the BLS can obtain biotin from the host. The spectrum of ABC transporters was similar in the BLS and other related members of Bartonellaceae (Fig. S7A). The ABC transporters included proteins involved in phosphate (*pstS*, *pstC*, *sptA,* and *pstB*), D-methionine (metQ, metI, and tN), zinc (znuA, znuB, and znuC), lipoprotein (LolC, LolD, and LolE), heme (CcmA-C), lipopysacchaaride (*LptF*, *LptG,* and *LptB*), macrolide (*MacB*), and microcin C (*yejA, yejB*, *yejE,* and *yejF*) transport. Among ABC-2-type components without transport functions, we identified proteins involved in cellular division (*FtsX* and *FTsE*). Among the transporters, microcin C genes exhibited higher expression levels than the other transporters (Fig. S7B).

**Fig 4:**
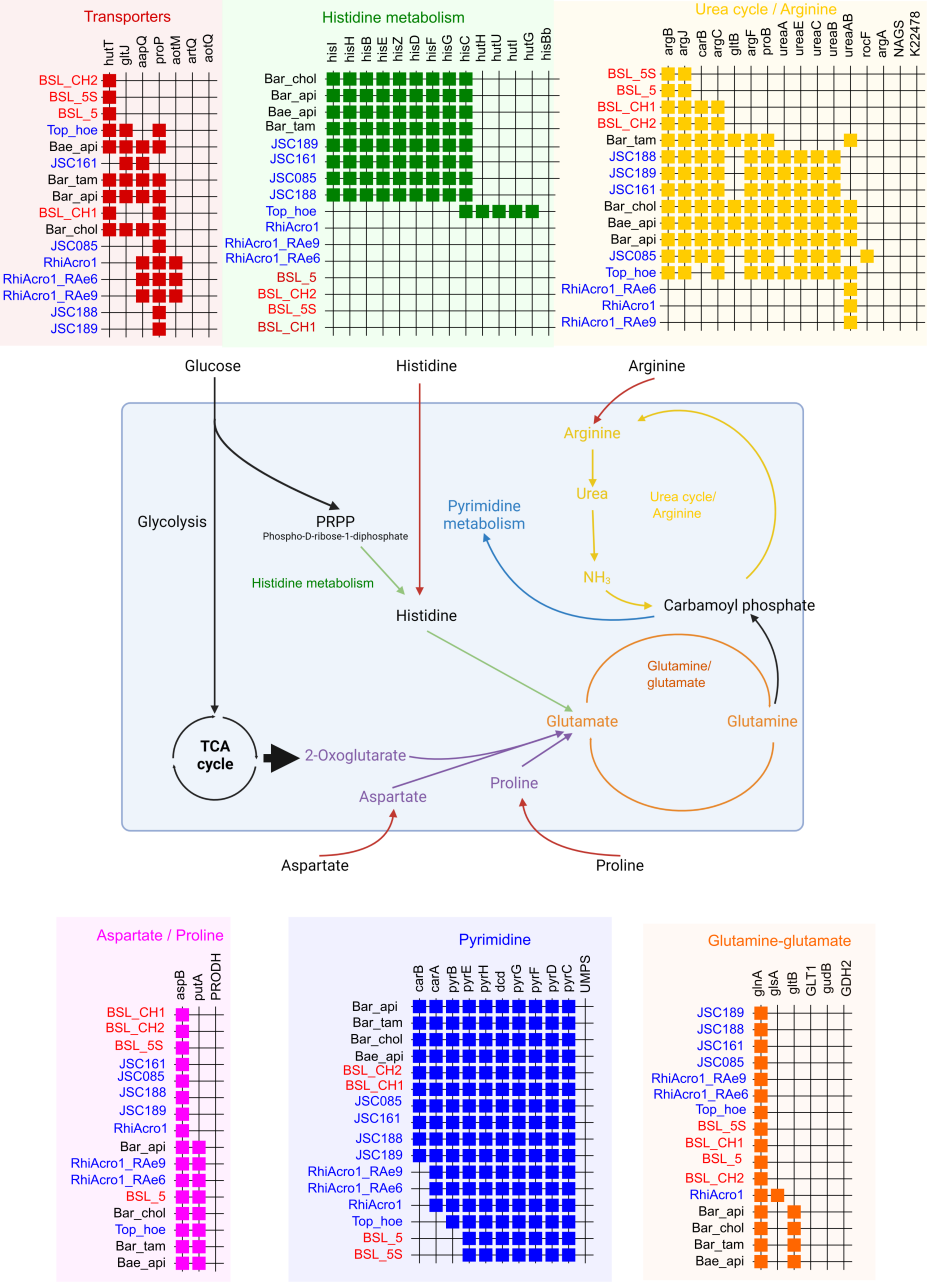
Biosynthesis and degradation of histidine and arginine in the *Bartonella*-like symbiont (BLS) and ant-associated *Tokpelaia* species. Data for *Tokpelaia* were modified from (11) to compare the selected transporters and enzymes involved in the import, synthesis and degradation of histidine and arginine, aspartate, and proline. The schema includes the glutamine/glutamate and arginine cycles. The seriation schema included comparison of the *Bartonella apis* group, *Ca*. *T. hoelldoblerii,* and *Ca*. Tokpelaia (RhiAcro1, RhiAcro1-RAEe6, RhiAcro1_Rae9) from *Acromyrmex* leaf-cutting ants (6) and then *Ca*. Tokpelaia (JSC161, JSC085, JSC188, JSC189) from *Dolichoderus* species (11). The BLS is indicated in red, and ant symbionts are indicated in blue.

*Ca*. Tokpelaia can convert arginine to urea and L-ornithine using arginase (6); however, this gene is absent in the BLS (Table S12). The BLS has complete pathways for threonine biosynthesis, glycine cleavage, lysine metabolism, ornithine biosynthesis, proline degradation, glutathione biosynthesis, and pyrimidine metabolism (Fig. 4), but the urea/arginine and histidine metabolism pathways are incomplete in the BLS.

Bartonellaceae has a group of horizontally transferred agents (BaGTA) of phage origin (34, 35). This group includes 14 proteins in *Bartonella australis* NH1 (35), and 29 CDs of the BaGTA locus were identified in *Bartonella henselae* (36). In the BLS, we identified phage 11 genes (Fig. S8 to S14), encoding lysozyme (*bgtA*), phage tail collar domain (*bgtC*), and phage tail protein (*bgtD*, *bgtF*, *bgtG*, *bgtH*, *bgtI*, *bgtJ*, *bgtK*, *bgtS,* and *bgtT*; while *bgtE* was missing). The presence of BagTA in both the BLS symbiont and *Ca*. Tokpelaia indicate the occurrence of а single HGT event and thus common ancestry for *Bartonella*, which is also supported by the fact that in *B. apis* groups, BaGTAs clustered separately (Fig. S7 to S14). Over the course of evolution, gene loss also occurred, e.g., in the BLS, *Ca*. Tokpelaia, and *Dolichoderus* ants (JSC161, JSC085, JSC188, JSC189), which showed the largest BaGTA gene reduction.

## DISCUSSION

In this study, we assembled the genomes of BLSs of *T. putrescentiae* (BLS_CH1; BLS_CH2; BLS_5; BLS_5S). The genomes shared a high identity, indicating that there was one type of symbiont among the different cultures of this mite. The 16S RNA gene of the BLS shared 98% similarity with previously identified 16S RNA genes from *T. putrescentiae* (12, 13, 25, 37, 38). Based on 16S rRNA gene comparison (Fig. 1), we found that the novel BLS is not restricted to *T. putrescentiae* and also occurs in other stored-product and house dust mites, such as *A. siro*, *C. lactis*, *D. farinae*, and *Dermatophagoides pteronyssinus*; this was corroborated by analysis of 16S rDNA clones, e.g., KM464397 and JX064706 (Table S8), and barcoding data (12–14, 39, 40). Our data also supported the existence of a novel clade of arthropod symbionts sister to the ant symbionts *Ca*. Tokpelaia (6, 10). Genome size reduction and gene loss have also been detected in *Bartonella* and *Ca*. Tokpelaia compared with their free-living relatives in Rhizobiales (11).

The BLS infection rate in mites was not 100% but started from 56%, indicating that this is a secondary symbiont (41). After recalculation, one mite was found to harbor 10^3^ BLS cells in its body; honeybees, for example, can host 10^7^ bacterial cells (42). In accordance with previous results, the BLS was present in the feces but not in the eggs (38). The low numbers of BLS copies observed (10^1^–10^2^) in some egg samples suggest the occurrence of contamination due to insufficient surface sterilization of the eggs. Thus, the BLS was transferred to the new mite host horizontally through the environment (feces, diet), not via a transovarial mode of transmission. The presence of the BLS in the feces indicated the presence of the bacterium in the gut. It has been suggested that *Ca*. Tokpelaia forms biofilms in the hindgut of ants (43). However, in mites, the hindgut is reduced, and bacteria are mostly found in the postcolonic diverticula of the midgut (44), attaching to the microvilli of the midgut cells (12). In the gut, bacteria most likely disperse via flagellar mobility prior to their attachment to host cell midgut cells.

It is believed that *Ca*. Tokpelaia contribute to the dietary switch from predatory to herbivory in ants (5). Our data did not indicate that the BLS contributes significantly to nitrogen recycling in mites. In addition, the urea/arginine and histidine metabolism pathways were also incomplete in the BLS. However, we observed that BLSs provide pantothenate and lipoic acid to the host. Previous experiments showed that the BLS proportion in the microbiome of *T. putrescentiae* increased after nutrient (25) or pesticide (26) stress. An analogous situation was observed with *B. apis* and honeybees, where the proportion of *B. apis* showed different responses to environmental stress (45–47). These studies support the hypothesis that BLSs are biologically important symbionts for their hosts (25–28).

Gram-negative bacteria export microcin to the environment to reduce competition from other bacterial strains (48). Microcin C consists of a nonhydrolyzable aspartyl–adenylate that is efficiently imported into bacterial cells. Inside the cell, the carrier is removed by proteolytic processing to release a potent aspartyl tRNA synthetase inhibitor (49). Although the main producers are Enterobacteriaceae, microcin operons were identified in *Bartonella* (48); however, in *Bartonella quintana* and *B. henselae,* microcin production is inactivated (50). The microcin C transporter is present in the *B. apis* group, *Ca*. *T. hoelldoblerii* and *Ca*. Tokpelaia from *Acromyrmex* and was found in the BLS. This finding suggests that the symbiont uses microcin to compete with other gut bacteria, which needs to be experimentally verified.

In conclusion, we identified an obligatory symbiont from the stored-product mite *T. putrescentiae*. The symbiont seems to be transmitted by the oral-fecal route. Genome reduction indicated that the symbiont is dependent on host metabolism while providing vitamins to its host. The population of the symbiont varied among mite cultures and individuals. The analyzed mite cultures originated from different stored product sources and different geographical location (Table 2).

## MATERIALS AND METHODS

### Samples of mites and feces

Cultures of *T. putrescentiae* (Table 2; Table S1) were maintained at the Crop Research Institute, Prague, Czechia, and in a laboratory in China as described previously (40, 51). Mite cultivation was performed in Iwaki flasks on a house dust mite diet (SPMd) (52). Mites were collected with a brush and placed into sterile tubes and weighed. For the experiments, we used 30–40 mg of mites weighed using a microbalance (Metler-Toledo). The fresh weight of mite is between 4–6 µg.

The spent growth medium (SPGM) was the fraction containing the diet debris, feces, and mite debris obtained from the rearing chamber; after this fraction was obtained, residual mites and/or eggs were removed (53). The weight was the same as that of fresh mites. The eggs were collected according to a protocol described previously (20). The samples for single mites were analyzed according to a previously published protocol (20, 54).

The mite population-level samples were processed by surface sterilization on ice. The mite surfaces were cleaned by placing them in 100% ethanol, followed by vortexing for 5 s and centrifugation at 13,000 × *g* for 1 min. The supernatant was replaced with a bleach solution containing 0.5% sodium hypochlorite, and the samples were then mixed by vortexing for 5 s and centrifuged at 13,000 × *g* for 2 min. The bleach was replaced by ddH_2_O, and this step was repeated twice to remove residual bleach. Mite population-level samples were used for genome and transcriptome analyses and qPCR, and SPGM and egg samples were used for qPCR.

### Sample processing

Transcriptome and genome samples were prepared as described previously (55). All samples were homogenized for 30 s in a glass tissue grinder (Kavalier glass, Prague, Czechia) in 500 µL of lysis buffer on ice. A NucleoSpin RNA kit (catalog no. 740984.50; Macherey-Nagel, Duren, Germany) was used for RNA extraction, with the following modifications: homogenized samples were centrifuged at 2,000 × *g* for 3 s, and DNA was degraded by DNase I at 37°C according to the manufacturer’s protocol (Riboclear plus, catalog no. 313-50; GeneAll, Lisbon, Portugal). RNA quality was evaluated using a NanoDrop instrument (NanoDrop One; Thermo Scientific, Waltham, MA, USA) and an Agilent 2100 Bioanalyzer (Agilent Technologies, Santa Clara, CA, USA). DNA was extracted from the homogenates after overnight incubation with 20 µL of proteinase K at 56°C using the QIAamp DNA Micro Kit (Qiagen, Hilden, Germany, cat. No. 56304) and following the manufacturer’s protocol for tissue samples. The concentration of the extracted DNA samples was quantified using a Qubit dsDNA HS Assay Kit (Life Technologies), and the quality of the DNA was determined using a NanoDrop 2000 instrument. The average gDNA size was determined using an E-Gel SizeSelect 2% Agarose Gel (Invitrogen) with a 1 kb ladder. The samples were sheared using a Covaris G-tube (Covaris Inc.). The average size of the sheared DNA was determined using a TapeStation 4200 system (Agilent Technologies).

### Genome assembly

For Illumina DNA sequencing, paired-end libraries were prepared using a Nextera DNA Flex library preparation kit (Illumina) and sequenced with 500 cycles using a NovaSeq 6000 system (Illumina). For PacBio sequencing, the library was prepared with the SMRTbell Express Template Prep Kit 2.0 (Pacific Biosciences) and sequenced on a PacBio Sequel system (Pacific Biosciences). The SMRT Link Circular Consensus Sequencing workflow (SMRT Link v.9.0.0, CCS) was used to combine multiple subreads from the same molecule to generate a highly accurate consensus sequence (see Table S1).

Illumina DNA reads were trimmed with Trim Galore (56) and processed using fastQC (57). Then, the reads were mapped onto reference data sets using Bowtie2 (58, 59) and Minimap2 (60) for long sequences. Our bacterial reference data set contained *Bartonella* genomes, *Ca*. Tokpelaia genomes, and a *Ca*. *T. hoelldoblerii* genome. The draft sequences were *de novo* assembled using SPAdes v3.13.1 in the metagenomic mode and further scaffolded using SSPACE Basic v2.0 with paired-end NGS short reads and SSPACE LongRead v1.1 with TGS long reads (61). Finally, after gap filling with GapCloser v1.12 (62) (in SOAPdenovo package) and Sealer (under ABySS v2.2.5) (63) using all the host removed NGS short reads, the genome was assembled in a single scaffold comprising four contigs. The assembled genome was polished using Pilon (64). Altogether, four genome assemblages were obtained: (i) single-contig annotation of the Chinese strain and (ii) multiple-contig annotation of the Chinese strain, both based on pacbio and illumina sequences from Chinese culture of *T. putrescentiae* (Table S1); (iii) multiple-contig annotation of a mix of *T. putrescentiae* cultures based on illumina sequences; and (iv) multiple-contig annotation of 5 S *T*. *putrescentiae* strain. No *Bartonella*-like sequences were obtained from PacBio sequences from mixed cultures (Table S1).

Bacterial sequences were annotated by Prokka (65) using DFAST (66) on the web server, and predicted proteins were identified by KEGG using GhostKoala (67). Predicted proteins were assigned to KEGG categories, and metabolic pathways were identified using KEGG Mapper (68). Additional analysis was performed using EggNOG Mapper (69). The genome was visualized in Proksee (60). The genomes were compared using the MASH algorithm (70) in dRep (71). Then, the identified proteins from the genomes were compared pairwise using PHMMER (72), and identical proteins were suggested to have a score ≥100.

### Phylogenomics and molecular identification

The whole-genome and 16S RNA sequences of the BLSs were analyzed. The 16S RNA sequence selection was extended to the 16S rRNA of bacteria identified based on their high similarity using NCBI BLAST (73) (see Table S8 for list of compared 16S RNA sequences). Sequences were aligned by MUSCLE v5 (74), and a maximum likelihood phylogenetic tree was inferred in PHYML 3.0 (75) (GTR + G + I model) by bootstrapping (100 replicates). Whole-genomic taxonomic analyses were performed incorporating available *Ca*. Tokpelaia and selected *Bartonella* genomes and the genomes of some members of Rhizobiales using the MASH algorithm (70) in dRep (71)(see Table S8 for list of compared taxa). Then, a set of selected genomes was compared to M1CR0B1AL1Z3R (https://microbializer.tau.ac.il) by detecting open reading frames (ORFs), finding orthologous groups, aligning orthologous sequences (76, 77), and inferring a maximum likelihood phylogenetic tree using RAxML with 100 bootstrap replicates (78). The genomes analyzed in M1CR0B1AL1Z3R are listened to in Fig. 1B and Table S8. All trees were rooted and visualized in FigTree.

The protein *Bartonella* gene transfer agent (BaGTA) was identified by pBLAST and PHMMER (79) using the BagTA database from *B. henselae* (36). Proteins were aligned in T-Coffee (80), and trees were inferred using PHYML and visualized in FigTree.

### Transcriptome analyses

Transcriptome analyses were performed for population-level mite samples from the 5K, 5L, 5N, 5P, 5Pi, 5S, and 5Tk cultures (Table 2), with seven replicates per culture (Table S1). Briefly (22), poly-A selection and library preparation were performed by using KAPA mRNA HyperPrep Kits (Roche), and paired-end sequencing was performed for 500 cycles using a NovaSeq 6000 system (Illumina). Read processing were performed as described previously (55), and RNA sequences were deposited in GenBank (Table S1). Transcriptome analyses of BLS_CH2 were performed in CLC Workbench 22 (Qiagen, Venlo, Netherlands). The number of mapped reads to BLS_CH2 was used as an indicator of expression.

### Quantification of *Bartonella*-like symbionts in mite cultures

The following cultures were used for BLS quantification (5K, 5L, 5N, 5P, 5Pi, 5S, and 5Tk) (Table 2). The quantification was based on the detection of BLSs in single mites using 30 mites and PCR. The next step was the quantification of the number of BLS copies by qPCR on population-level samples of mites, eggs, and SPGM.

PCRs for individuals were carried out according to a protocol described previously (54) using the *Bartonella*-specific primers Bart_1 F x Bart_1R (specific for *Bartonella*-like 16s DNA clones from mites) (12) (Table 2). Briefly, each reaction contained 12.5 µL of EmeraldAmp MAX HS PCR 2× Master Mix (Catalog number RR330A, TaKara, Kyoto, Japan), 8.5 µL of dH2O, 0.4 µM each primer, and 2 µL of mite lysate, with a total reaction volume of 25 µL. Every PCR run contained a positive control (genomic DNA) and ddH2O (negative control).

Amplification by qPCR was carried out in a StepOnePlus Real-Time PCR System (Life Technologies, Grand Island, NY, USA) in 96-well plates using GoTaq qPCR Master Mix (Promega). SYBR Green (Bio-Rad Laboratories, Veenendaal, Netherlands) was employed as a double-stranded DNA (dsDNA) binding dye according to a previously described protocol (20, 27, 81) with the bacterium-specific primers Bart_1Fq 5-GAATGTTAGCCGTTGGTAGG-3 and Bart_1R 5-GCAGCACCTGTCTCCGAC-3, amplifying 200 bp of the 16S rRNA. The standards were prepared from *Bartonella*-like bacterial clones obtained previously (81). Amplification was performed as follows: hot start activation with 1 cycle at 95°C for 60 s; 40 cycles of denaturation at 95°C for 15 s; and annealing at 62°C for 30 s; melt curve analysis at 95°C for 15 s, 60°C for 30 s, and 95°C for 15 s. All reactions were conducted in technical duplicates for six biological replicates per mite culture, mite body, SPGM, and egg sample. Microbial gene abundance was normalized to per-mite, per-egg, and per-gram of SPGM values. Before analyses, gene abundance data were log (10)-transformed. Abundance values lower than the detection limit were replaced by zeros.

### Biotest

To test the effect of folic acid and pantothenate on mite growth, the two vitamins (cat Nos 1912 and 3821 Carl Roth GmbH & Co. KG, Karlsruhe, Germany) were added separately to crushed oat flakes at 1% dry weight as described previously (82). Briefly, the diet (0.1 ± 0.01 g) was transferred into IWAKI chambers in six replicates per mite culture. The control was a chamber containing the diet without folic acid. Ten adults were added to every chamber, and the chambers were incubated at 85% RH and 25 ± 0.5°C for 21 days. After the cultivation period, the chambers were filled with 50 mL of Oudemans solution (70% ethanol, acetic acid, and glycerol; 87:8:5, vol/vol/vol), and mites were counted under a dissection microscope.

### Statistical analyses

Gene expression analyses were performed in R using the vegan package (83). The variable “mite culture” was tested as an environmental variable in dbRDA analyses based on Jaccard and robust Aitchinson distances. Both similarity distances need no data standardization. Gene expression was tested for all genes, and genes were assigned to KEGG pathways. The mite population density in the biotest was influenced by mite culture in the control treatment. Therefore, the data in the experiment were divided by the main density of mites in the culture. Then, the data were analyzed using the nonparametric Kruskal–Wallis test, and the differences among the mite cultures with high and low densities of BLSs were analyzed using the Mann–Whitney test, all calculated in PAST 4 (84).

## SUPPLEMENTAL MATERIAL

Supplemental Figures msystems.00829-23-s0001.docx Figures S1-S19.

Supplemental Tables msystems.00829-23-s0002.xlsx Tables S1-S14.

ASM does not own the copyrights to Supplemental Material that may be linked to, or accessed through, an article. The authors have granted ASM a non-exclusive, world-wide license to publish the Supplemental Material files. Please contact the corresponding author directly for reuse.
