# Supplementary material for: A novel Bartonella-like bacterium forms an interdependent mutualistic symbiosis with its host, the stored-product mite Tyrophagus putrescentiae
Source: mSystems. 2024 Feb 21;9(3):e00829-23. doi: 10.1128/msystems.00829-23 (PMC10949449; doi:10.1128/msystems.00829-23)

**Supplementary Figures S1–S19**

**Title:** A novel *Bartonella*-like bacterium forms an interdependent mutualistic symbiosis with its host, the stored-product mite *Tyrophagus putrescentiae*

**Authors:** Q. Xiong, B. Sopko, P. B. Klimov, J. Hubert

**Journal:** mSystems

Note: BLS - *Bartonella*-like symbiont

**Fig S1** Comparison of the genome assemblies of *Bartonella*-like symbionts of *Tyrophagus putrescentiae*. **A** – The protein comparison among genomes is shown as Venn diagrams. The proteins were compared pairwise by PHMMER, and those with scores higher than 100 were suggested to be identical for Venn diagram construction. **B** – Comparison of the genomes based on the proteins assigned to KEGG pathways using a Venn diagram. **C** - Comparison of genome similarity based on MASH clustering using dREP.


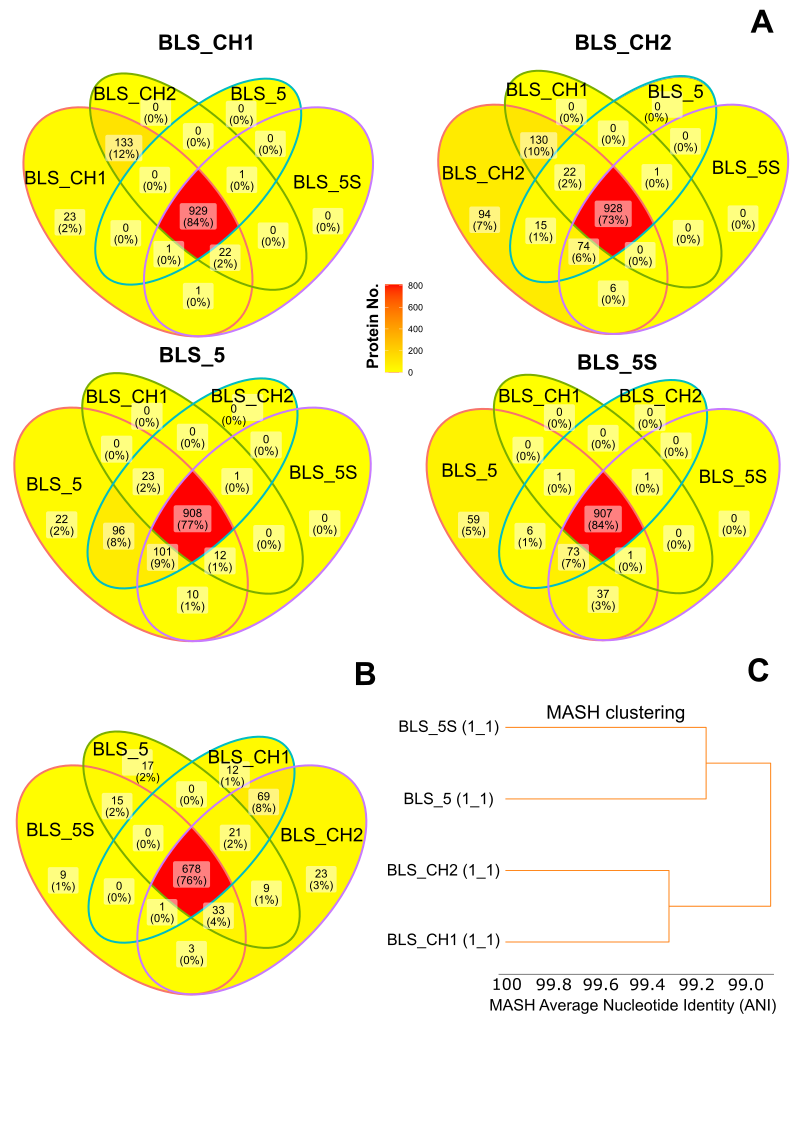


**Fig S2** OFR counts and GC contents of the compared relevant genomes of *Bartonella* clade and BLS_CH2 (red dot) in M1CR0B1AL1Z3R.


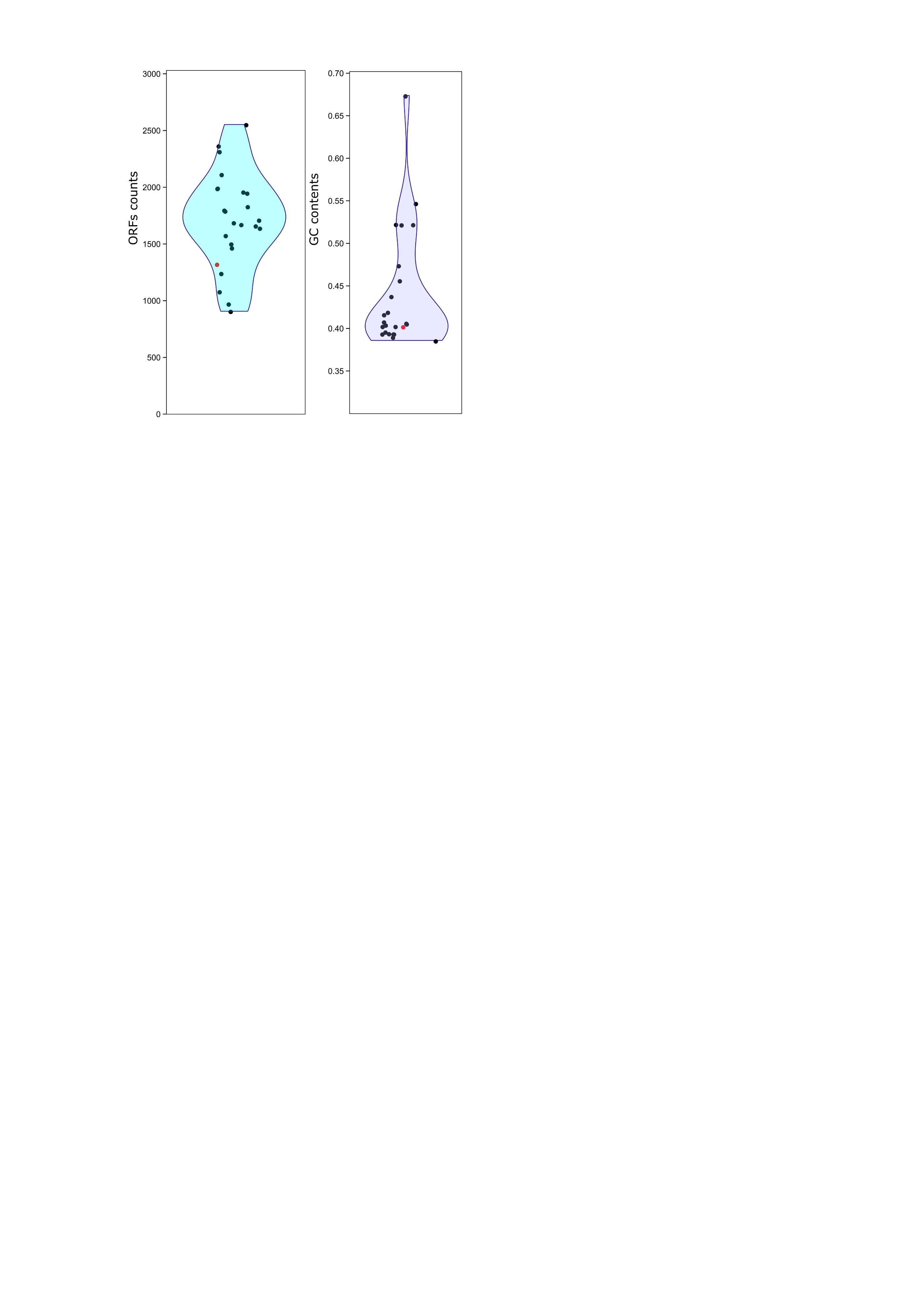


**Legend:** The taxa from *Bartonella* clade are listed in Fig. 1B and Supplementary dataset Table S8.

**
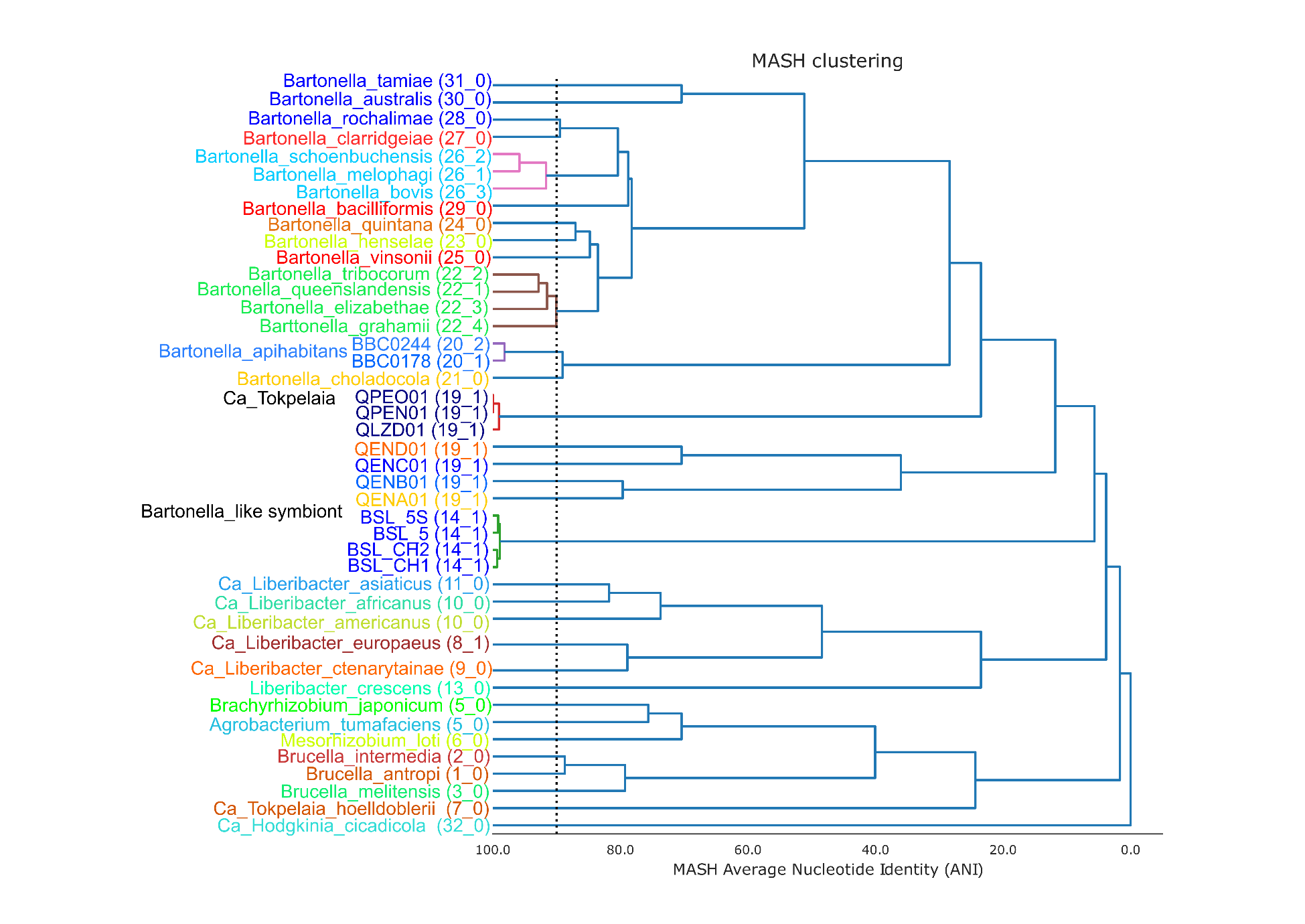
Fig S3** Comparison of *Bartonella*-like symbionts *of Tyrophagus putrescentiae* to selected Bartonellaceae genomes, available *Ca*. Tokpelaia and some members of Rhizobiales (Supplementary dataset: Table S8); genome similarity was determined based on MASH clustering using dRep.

**Fig S4** Comparison of *Bartonella*-like symbionts of *Tyrophagus putrescentiae* BLS_5CH2 proteins against members of *Bartonella* clade: *Bartonella tamiae*, *Bartonella choladocola*, *Ca.* Tokpelaia hoelldoblerii, and *Ca.* Tokelaia (QPEN01, QLZD01, QPWO01, QENA01, QENB01, QEN01 and QEND01). The comparison is based on the proteins identified by PHMMER, and the data of selected Bartonellaceae were used individually. A protein match with a score higher than 100 was considered identical for Venn diagram construction **A** and seriation **B**.


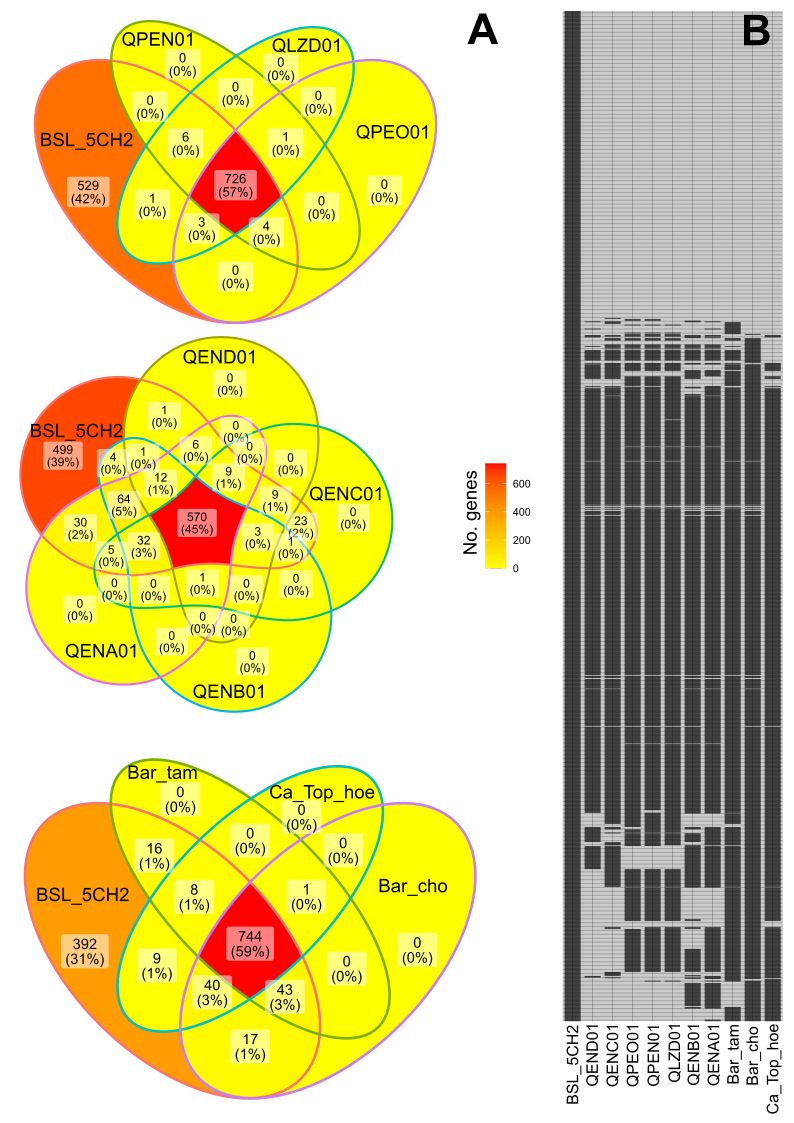


**Fig S5** Quantification of the *Bartonella*-like symbionts in selected *Tyrophagus putrescentiae* 7 mite cultures. **A** – ordiplot of dbRDA of gene expression in BLS_CH2 in mite transcriptome samples; mite cultures are shown as hulls, while important expressed genes are shown by arrows; negative CAP1 values represent cultures with higher expression (5P, 5K and 5N); **B** – of BLS_CH2 in mite transcriptome samples, boxplots show numbers of reads after log 10 transformation; **CD** 16S qPCT abundance estimates of BLS based on specific primers, boxplots are numbers of reads after log 10 transformation; **C** –mite bodies**,** D fecal fraction (SPMG).


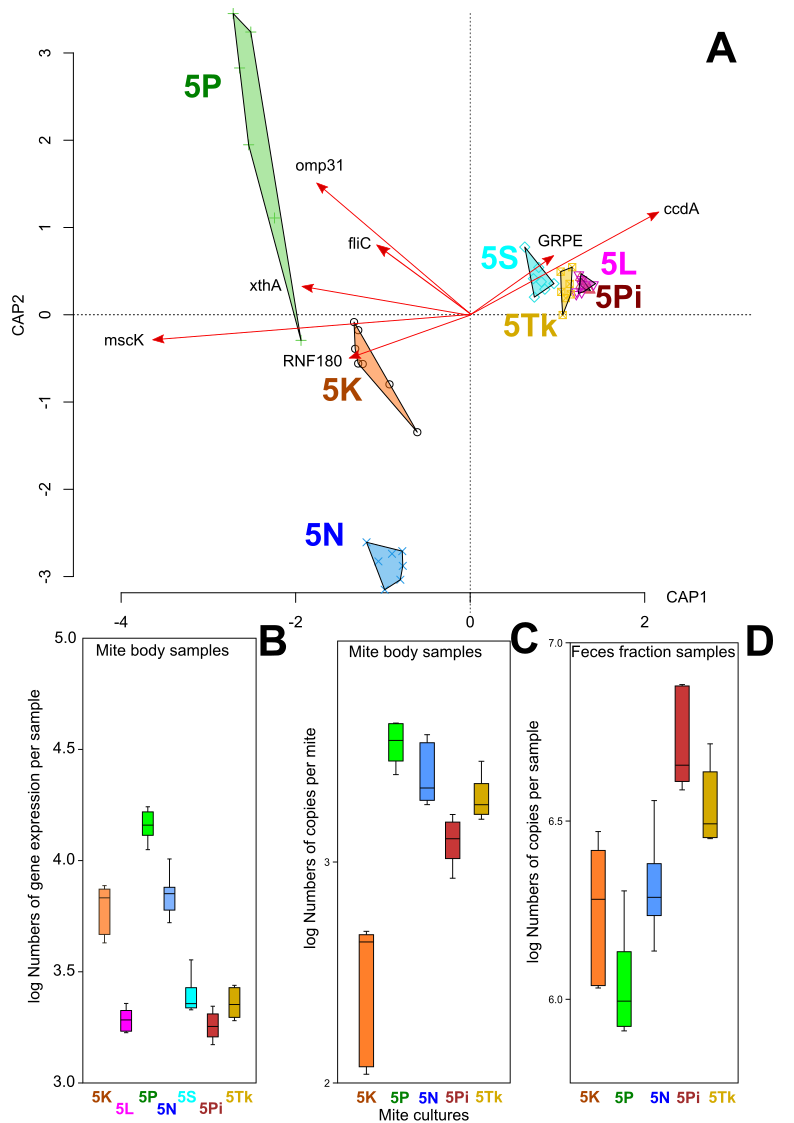


**Fig S6** Maximum likelihood tree (PHYML) of *TolC* (K12340) proteins based on T-coffee structural alignments. The numbers above branches are support values calculated from 100 pseudobootstrap replicates; the tree was rooted in *Brevundimonas halotolerans*. PHMMER-identified domain OEP 9 (outer membrane efflux protein domains), SP (signal peptide) and the positions of the key protein sequences and domains in these proteins are shown in the image. A jagged edge instead of a curved edge indicates that a sequence match does not pass through the first position.


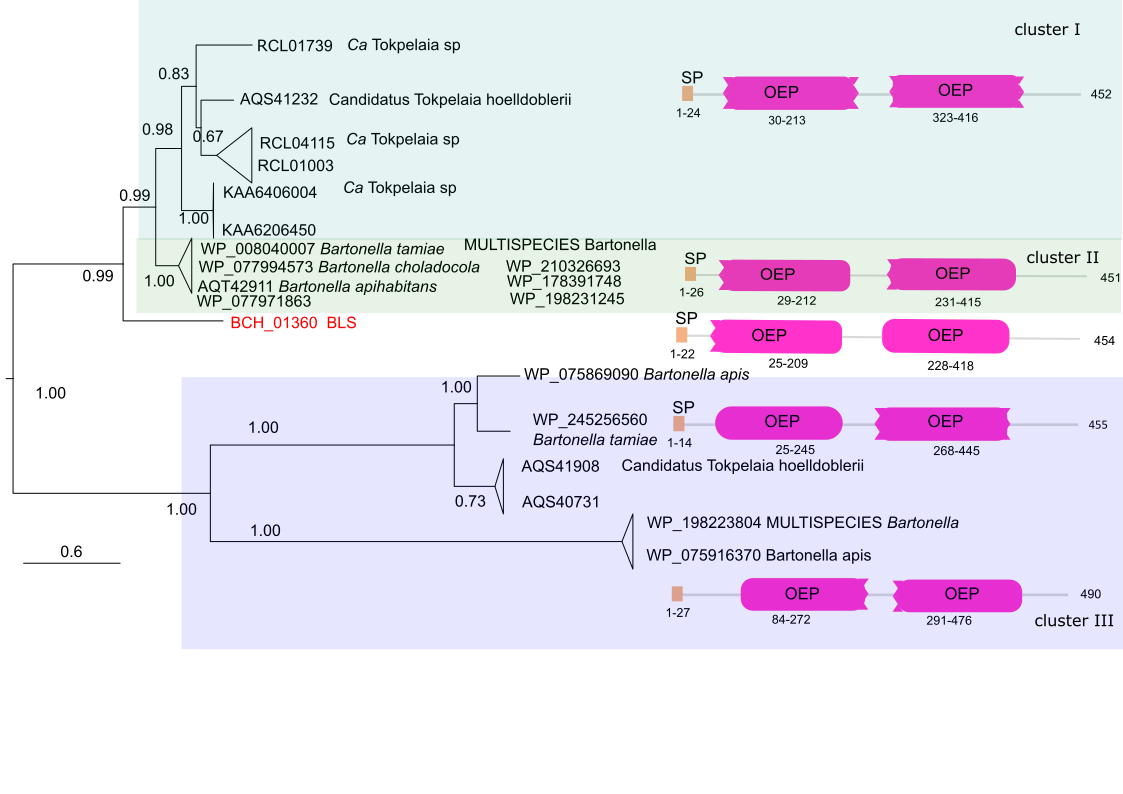


**FIG S7** KEGG genes of ABC transporters (**A**) and their expression in different mite cultures (**B**). **A** – Gene presence/absence in the *Bartonella*-like symbiont and related taxa based on seriation; different gene groups are color-coded; different isolation sources of *Ca*. Tokpelaia are indicated by blue colors in the labels. **B** – Gene expression in *Bartonella*-like symbionts from seven mite cultures.

**
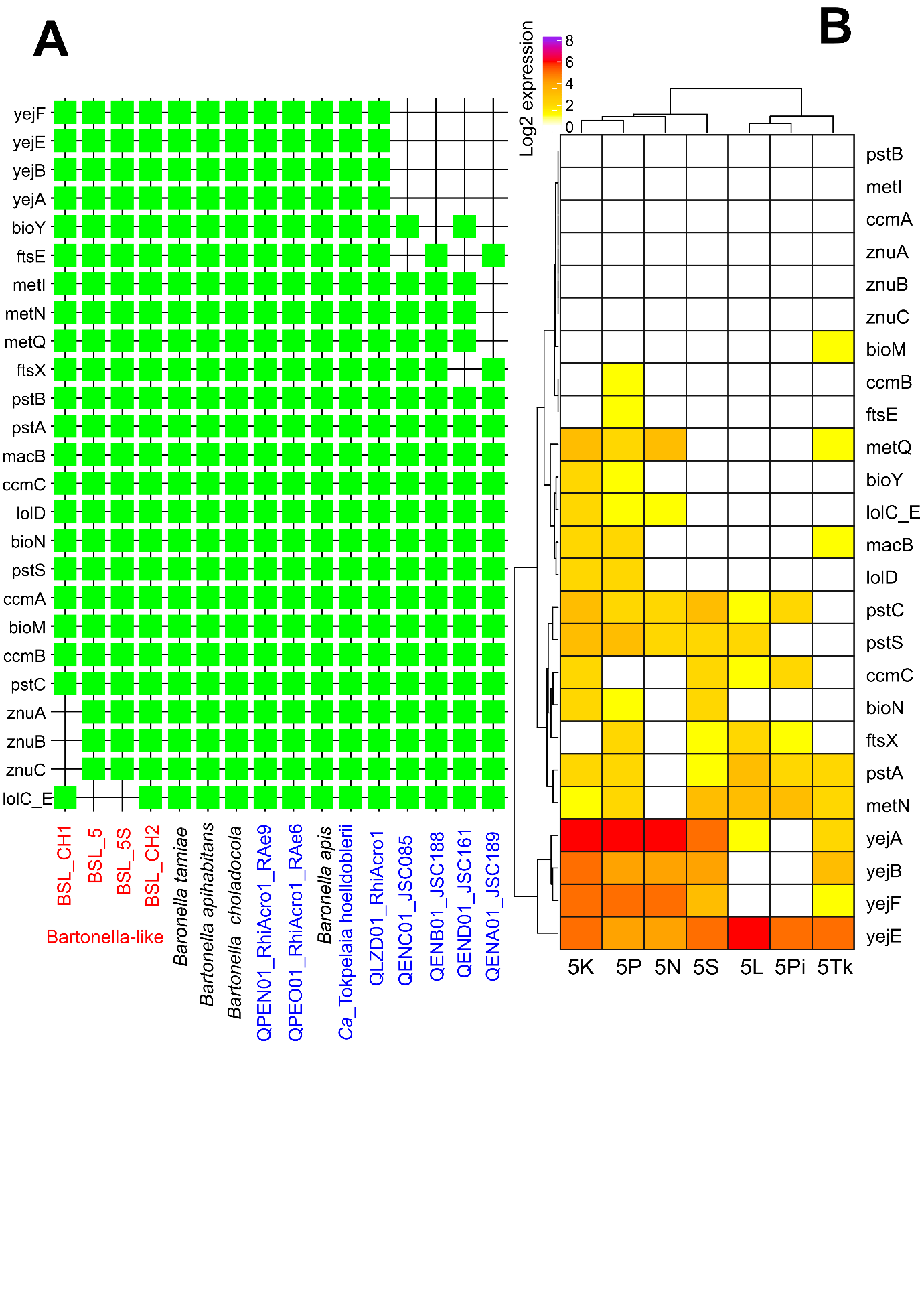
**

**Fig S8** *Bartonella* gene transfer agent (BaGTA), phage lysozyme *bgtA*. Data were aligned in T-coffee; tree inference was performed in PHYML (WAG+G+I); the numbers above branches are support values from 100 pseudobootstrap replicates; the tree was rooted on *Brucella* phage *BiPBO1* lysozyme (GenBank: ALJ98247). The BLS protein is marked in red, and *Ca*. Tokpelaia is indicated in blue.


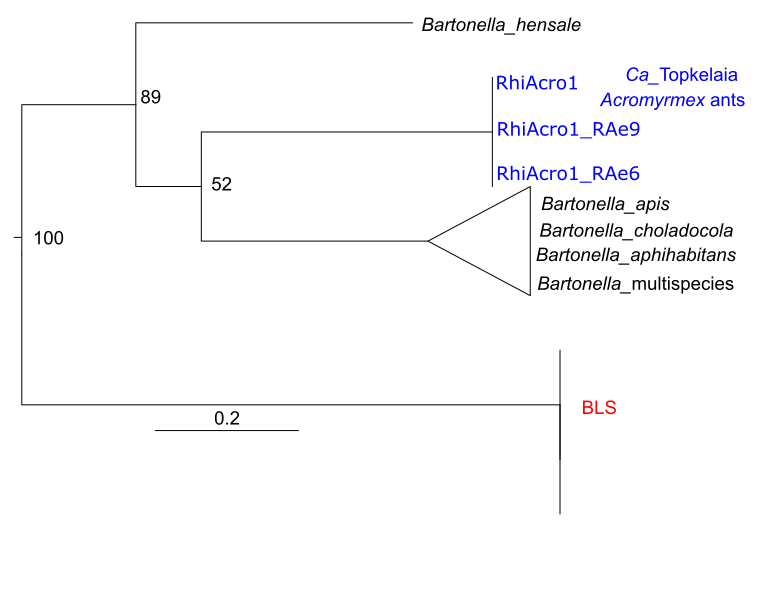


**Fig S9** *Bartonella* gene transfer agent (BaGTA), Phage Tail Collar Domain *bgtC***;** tree inferred with PHYML (MtREV +G+I) from T-coffee combined alignment. The numbers above the branches are pseudobootstrap support values from 100 replications, and the tree was rooted in the *Rhizobium subbaraonis* phage tail collar domain (GenBank SOC40100). BLS proteins are marked in red, and *Ca*. Tokpelaia is indicated in blue.


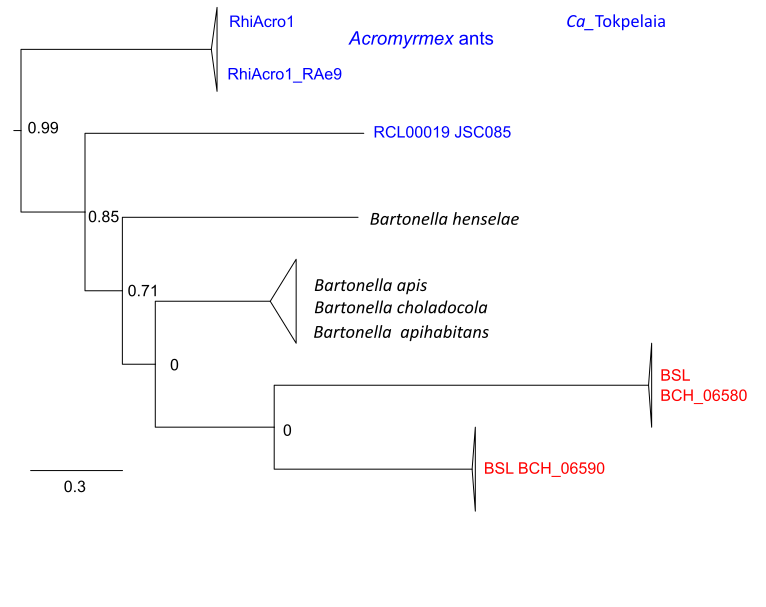


**Fig S10** *Bartonella* gene transfer agent (BaGTA), phage tail protein *bgtD*; tree inferred with PHYML (RtREV+G+I+F) from T-coffee combined alignment. The numbers above branches are pseudobootstrap support values from 100 replications, and the tree was rooted in *Falsochrobactrum ovis* (RAK29153). BLS proteins identified as *bgtD* are marked in red, and *Ca*. Tokpelaia is indicated in blue.


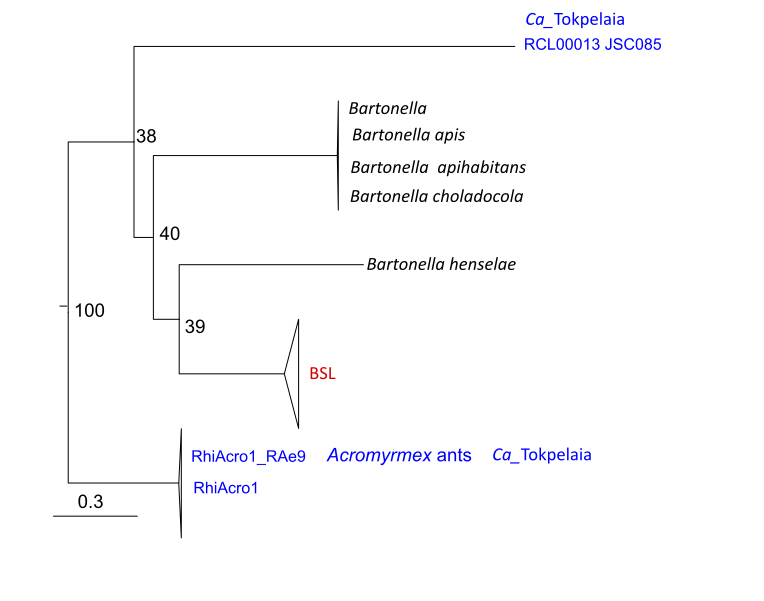


**Fig S11** *Bartonella* gene transfer agent (BaGTA), hypothetical protein *bgtE*; tree inferred with PHYML (Q, Pfam +G) from T-coffee combined alignment. The numbers above the branches are pseudobootstrap support values from 100 replications, and the tree was rooted in *Falsochrobactrum ovis* (RAK29149). BLS proteins identified as *bgtE* are marked in red, and *Ca*. Tokpelaia is indicated in blue.

**
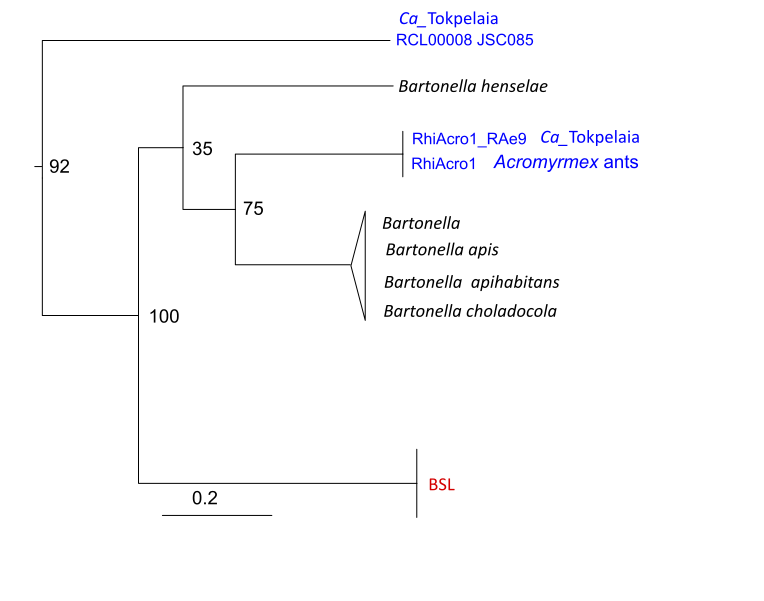
**

**Fig S12** *Bartonella* gene transfer agent (BaGTA), hypothetical protein *bgtF;* tree inferred with PHYML (Q, Pfam +G) from T-coffee combined alignment. The numbers above branches are pseudobootstrap support values from 100 replications, and the tree was rooted in *Methylobacterium* sp. Leaf399 (KQT07815). BLS proteins identified as *bgtF* are marked in red, and *Ca*. Tokpelaia is indicated in blue.


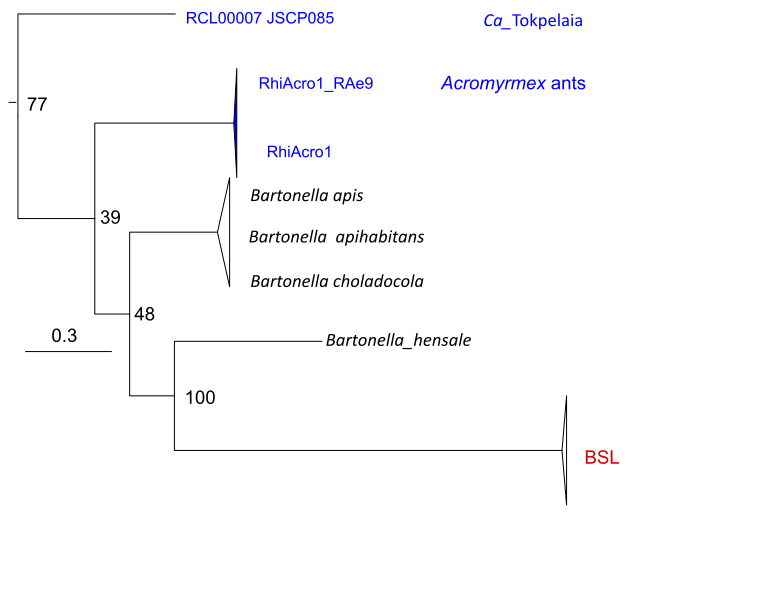


**Fig S13** *Bartonella* gene transfer agent (BaGTA), hypothetical protein *bgtG*; tree inferred with PHYML (Q, Pfam +G+I) from T-coffee combined alignment. The numbers above branches are pseudobootstrap support values from 100 replications, and the tree was rooted in *Azorhizobium caulinodan* (BAF86841). No match was found for *Bartonella choladocola* for *bgtG*. BLS symbiont proteins identified as *bgtG* are marked in red, and *Ca*. Tokpelaia is indicated in blue.


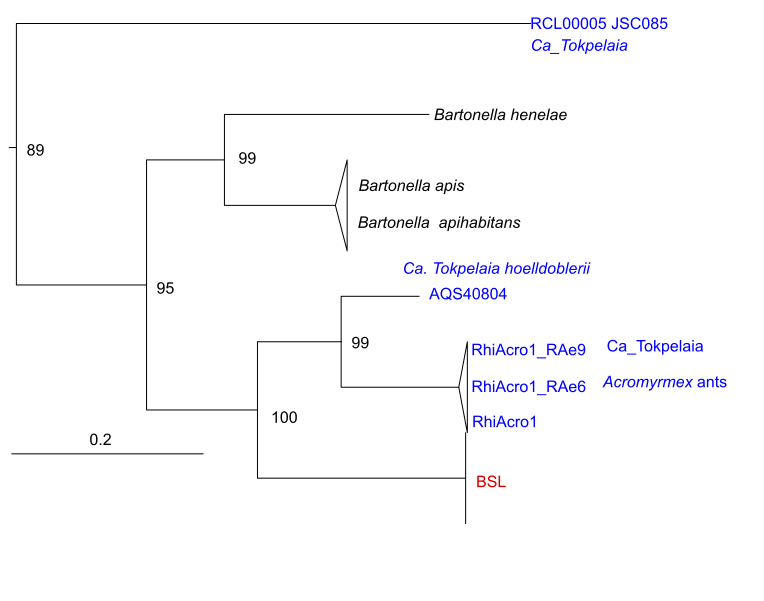


**Fig S14** *Bartonella* gene transfer agent (BaGTA), hypothetical protein *bgtH*; tree inferred with PHYML (Q,pfam+G+I+F) from T-coffee combined alignment. The numbers above branches are pseudobootstrap support values from 100 replications, and the tree was rooted in *Azorhizobium caulinodans* (BAF86839). BLS proteins identified as *bgtH* are marked in red, and *Ca*. Tokpelaia is indicated in blue.


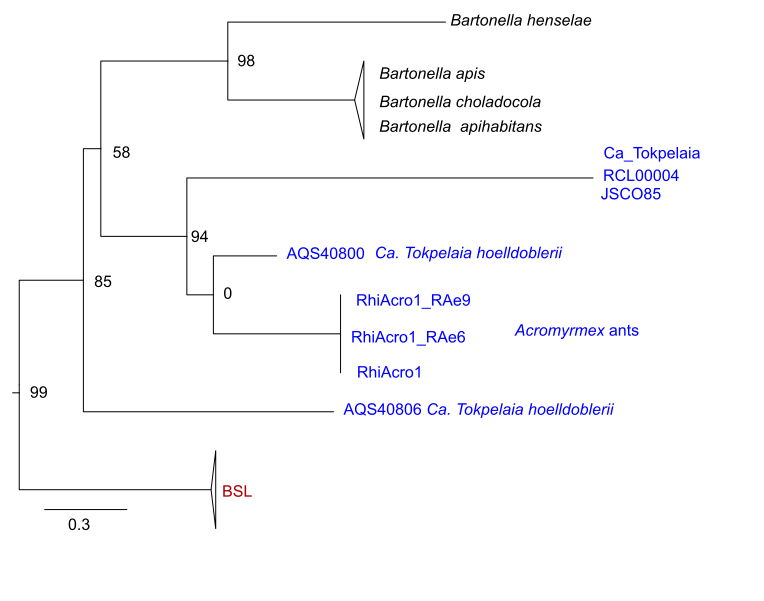


**Fig S15** *Bartonella* gene transfer agent (BaGTA), hypothetical protein *bgtI*; tree inferred with PHYML (Q, yeast +G+F) from T-coffee combined alignment. The numbers above branches are pseudobootstrap support values from 100 replications, and the tree was rooted in *Azorhizobium caulinodans* (BAF86838). BLS proteins identified as *bgtI* are marked in red, and *Ca*. Tokpelaia is indicated in blue.


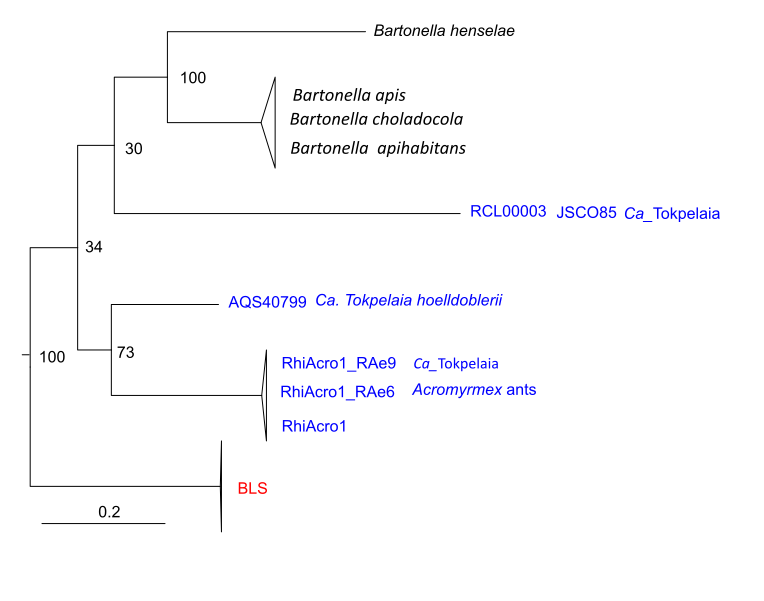


**F****ig S16** *Bartonella* gene transfer agent (BaGTA), hypothetical protein *bgtJ*; tree inferred with PHYML (Q, mammal+G+F) from T-coffee combined alignment. The numbers above the branches are pseudobootstrap support values from 100 replications, and the tree was rooted in *Rhizobium album* (PWE57143). BLS proteins identified as *bgtJ* are marked in red, and *Ca*. Tokpelaia is indicated in blue.


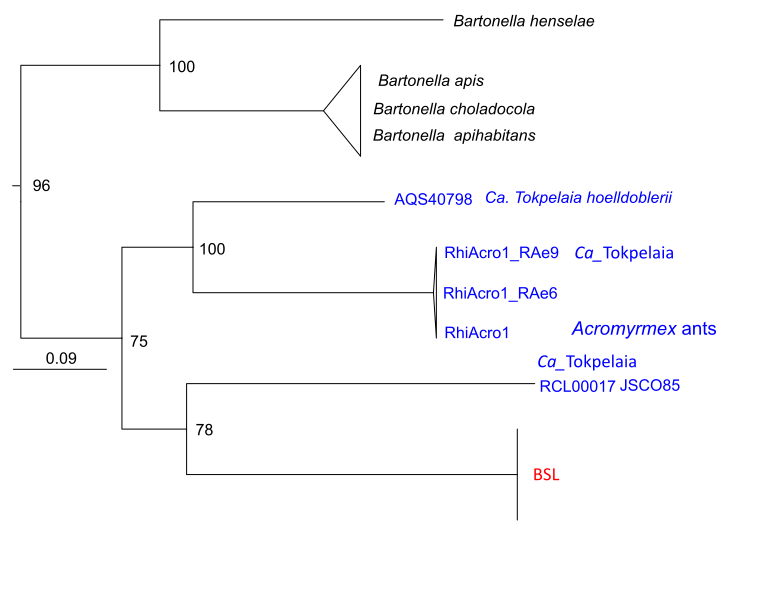


**Fig S17** *Bartonella* gene transfer agent (BaGTA), hypothetical protein *bgtK*; tree inferred with PHYML (JTT +G) from T-coffee combined alignment. The numbers above the branches are pseudobootstrap support values from 100 replications, and the tree was rooted in *Brucella thiophenivorans* (OYR13079). BLS proteins identified as *bgtK* are marked in red, and *Ca*. Tokpelaia is indicated in blue.


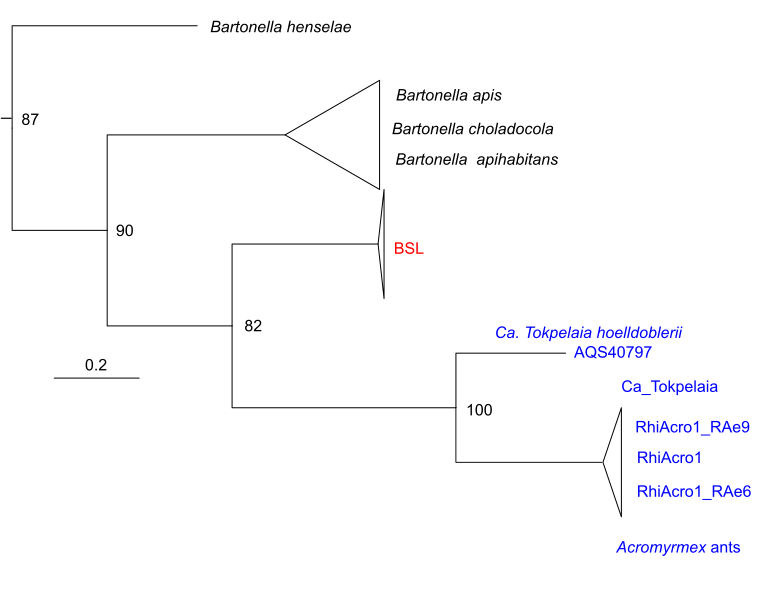


**Fig S18** *Bartonella* gene transfer agent (BaGTA), hypothetical protein *bgtS*; tree inferred with PHYML (LG +G+I) from T-coffee combined alignment. The numbers above the branches are pseudobootstrap support values from 100 replications, and the tree was rooted in *Rhizobium album* (PWE56781). BLS proteins identified as *bgtS* are marked in red, and *Ca*. Tokpelaia is indicated in blue.


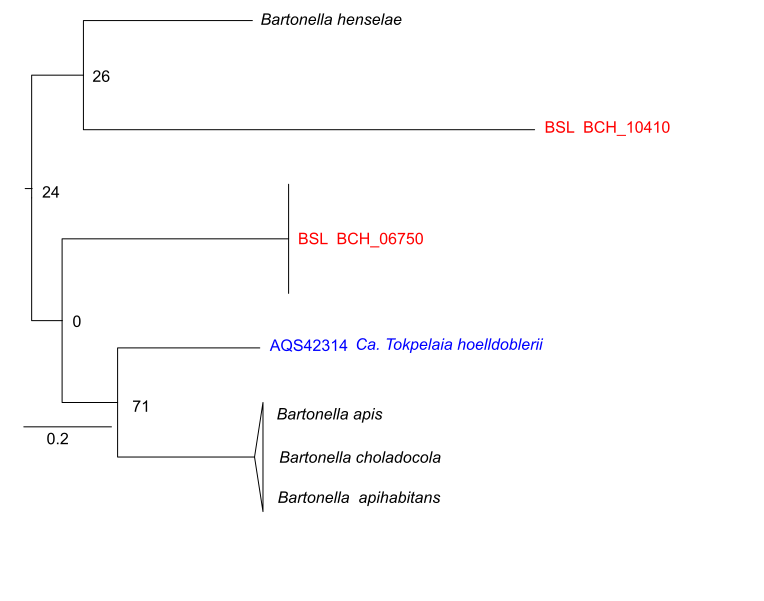


**Fig S19** *Bartonella* gene transfer agent (BaGTA), hypothetical protein *bgtT*; tree inferred with PHYML (Q, pfam +R) from T-coffee combined alignment. The numbers above the branches are pseudobootstrap support values from 100 replications, and the tree was rooted in *Brucella* phage BiPBO1 lysozyme (ALJ982470). BLS proteins identified as *bgtT* are marked in red, and *Ca*. Tokpelaia is indicated in blue.


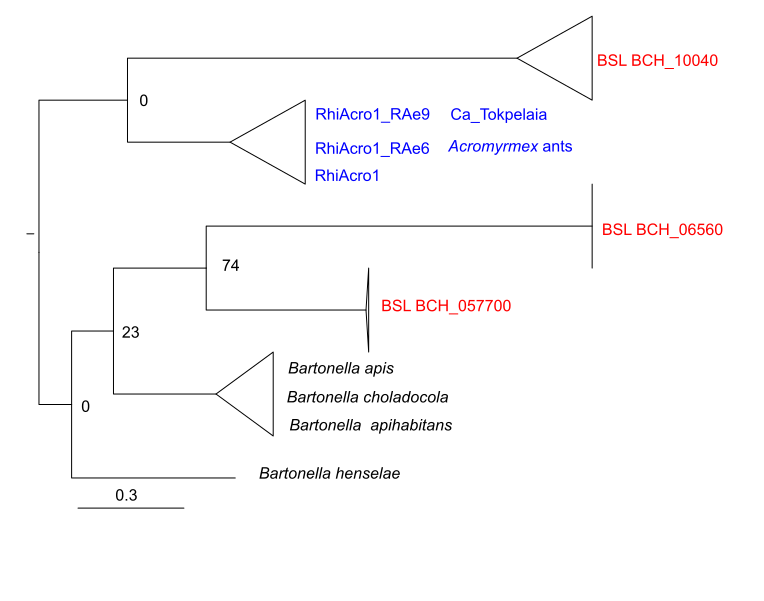

Supplement: Supplemental Figures — Figures S1-S19. [file msystems.00829-23-s0001.docx]
